# Supplementary material for: Plasmon Driven Nanocrystal Transformation by Aluminum Nano-Islands with an Alumina Layer
Source: Nanomaterials (Basel). 2023 Feb 28;13(5):907. doi: 10.3390/nano13050907 (PMC10005069; doi:10.3390/nano13050907)
Supplement: Supplementary file 1 [file nanomaterials-13-00907-s001.zip › nanomaterials-2207036-supplementary.pdf]

Supplementary Materials

Plasmon Driven Nanocrystal Transformation by Aluminum Nano-Islands with an Alumina Layer

Xilin Zhou [1], Huan Chen [1], Baobao Zhang [1], Chengyun Zhang [2,*], Min Zhang [1], Lei Xi [1], Jinyu Li [1], Zhengkun Fu [1,*] and Hairong Zheng [1]

[1] School of Physics and Information Technology, Shaanxi Normal University, Xi'an 710119, China; xilin@snnu.edu.cn (X.Z.); chenhuan@snnu.edu.cn (H.C.); bbzhang@snnu.edu.cn (B.Z.); minz@snnu.edu.cn (M.Z.); leixi@snnu.edu.cn (L.X.); 15035806369@163.com (J.L.); hrzheng@snnu.edu.cn (H.Z.)

[2] School of Electronic Engineering, Xi'an University of Posts & Telecommunications, Xi'an 710121, China

* Correspondence: cyzhang@xupt.edu.cn (C.Z.); zkfu@snnu.edu.cn (Z.F.)

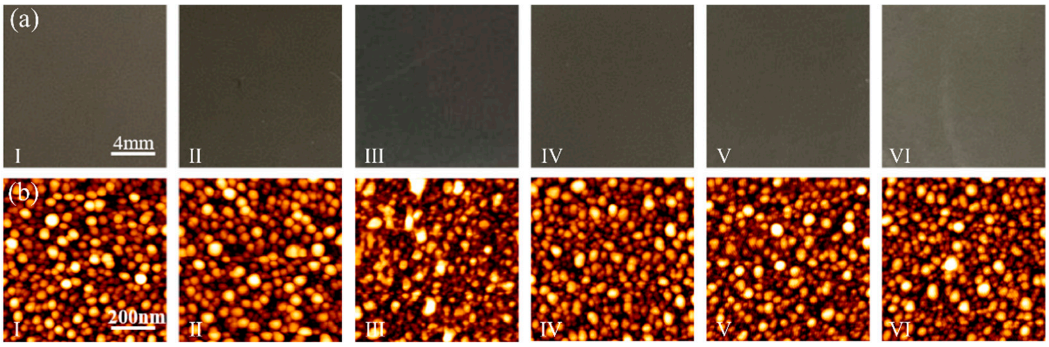

Figure S1. (**a**) Optical and (**b**) AFM images of the Al film without annealing (corresponding to I) and with annealing at 100 °C, 200 °C, 300 °C, 400 °C, 500 °C (corresponding to II-VI), respectively.

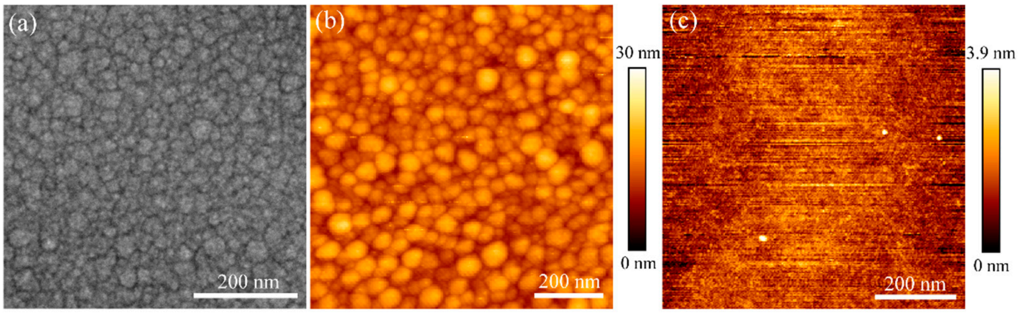

Figure S2. (**a**) SEM image and (**b**) AFM image of the Al/Al_2O_3 structure. (**c**) AFM image of 50 nm Al_2O_3 later.

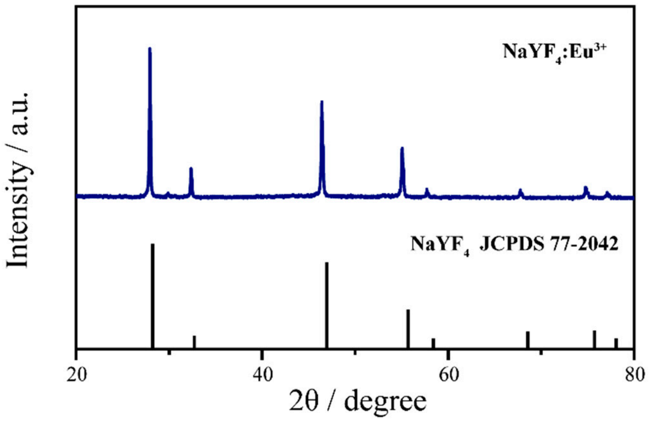

Figure S3. XRD pattern of the as-synthesized $NaYF_4:Eu^{3+}$ nanoflower and the standard pattern of cubic-phase $NaYF_4$ (JCPDS No.77-2042).

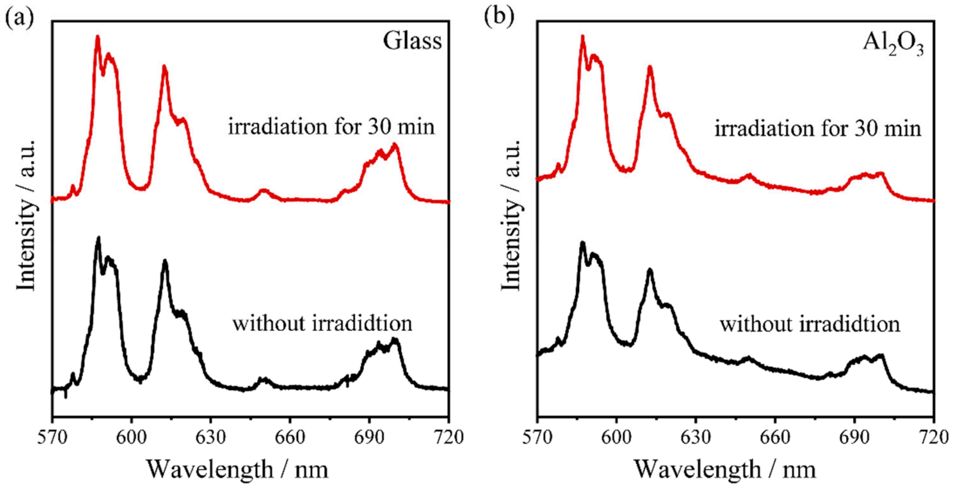

Figure S4. Luminescence spectra of $NaYF_4:Eu^{3+}$ (**a**) on a glass substrate without Al NIs and (**b**) with 50 nm Al_2O_3 deposited on a glass substrate before and after 976 nm (23 mW) laser irradiation for 30 min.
